# A pilot observational study of gait changes over time before and after an unplanned hospital visit in long-term care residents with dementia

**DOI:** 10.1186/s12877-023-04385-0

**Published:** 2023-11-08

**Authors:** Hoda Nabavi, Sina Mehdizadeh, Leia C. Shum, Alastair J. Flint, Avril Mansfield, Babak Taati, Andrea Iaboni

**Affiliations:** 1 KITE Research Institute - Toronto Rehabilitation Institute, University Health Network, 550 University Avenue, Toronto, ON M5G 2A2 Canada; 2 Department of Psychiatry, University of Toronto, Toronto, ON Canada; 3 Centre for Mental Health, University Health Network, Toronto, ON Canada; 4 Evaluative Clinical Sciences, Hurvitz Brain Sciences Program, Sunnybrook Research Institute, Toronto, ON Canada; 5 Department of Physical Therapy, University of Toronto, Toronto, ON Canada; 6 Department of Computer Science, University of Toronto, Toronto, ON Canada; 7 Institute of Biomaterials and Biomedical Engineering, University of Toronto, Toronto, ON Canada; 8 Vector Institute for Artificial Intelligence, Toronto, ON Canada

**Keywords:** Hospitalization, Nursing homes, Falls, Longitudinal analysis, Walking patterns, Computer vision

## Abstract

**Background:**

Older adults with dementia living in long-term care (LTC) have high rates of hospitalization. Two common causes of unplanned hospital visits for LTC residents are deterioration in health status and falls. Early detection of health deterioration or increasing falls risk may present an opportunity to intervene and prevent hospitalization. There is some evidence that impairments in older adults’ gait, such as reduced gait speed, increased variability, and poor balance may be associated with hospitalization. However, it is not clear whether changes in gait are observable and measurable before an unplanned hospital visit and whether these changes persist after the acute medical issue has been resolved. The objective of this study was to examine gait changes before and after an unplanned acute care hospital visit in people with dementia.

**Methods:**

We performed a secondary analysis of quantitative gait measures extracted from videos of natural gait captured over time on a dementia care unit and collected information about unplanned hospitalization from health records.

**Results:**

Gait changes in study participants before hospital visits were characterized by decreasing stability and step length, and increasing step variability, although these changes were also observed in participants without hospital visits. In an age and sex-adjusted mixed effects model, gait speed and step length declined more quickly in those with a hospital visit compared to those without.

**Conclusions:**

These results provide preliminary evidence that clinically meaningful longitudinal gait changes may be captured by repeated non-invasive gait monitoring, although a larger study is needed to identify changes specific to future medical events.

**Supplementary Information:**

The online version contains supplementary material available at 10.1186/s12877-023-04385-0.

## Introduction

Older adults with dementia have high rates of emergency department visits and hospitalization, some of which may be preventable. About 43% of older adults with dementia visit the emergency department every year and spent 2.5 h longer in Canadian emergency departments than those without dementia [1]. They also have 65% higher hospitalization rates and are more prone to hospital-related adverse events, such as delirium, falls, and infections, than other seniors [1]. The issue of potentially preventable hospital visits is particularly important for older adults residing in long-term care (LTC) homes [2, 3], most of whom have dementia [4]. About one-third of seniors living in LTC visit the emergency department each year, and one-third of these visits are potentially preventable [5]. The most common reason for hospital visits are a decline in medical status (e.g. fever, infection, shortness of breath) or a fall [6]. Preventable visits are associated with significant distress in older adults with dementia due to the unfamiliar and sometimes ‘chaotic’ environment, changes in their daily routine, and exposure to tests and treatments [7], and may have adverse consequences such as delirium or nosocomial infection. At a systems level, potentially preventable hospital visits by LTC residents represent a significant cost to the healthcare system and contribute to emergency department overcrowding and acute care bed shortages [8]. Developing innovative approaches to reduce the rate of preventable hospital visits is an important strategy to address this issue.

One possible approach involves the use of health monitoring technologies for early detection of a decline in health status or increase in falls risk. At present, identifying decline in health status of the residents of LTC is based on observation by healthcare staff, primarily personal support workers and nurses, who may or may not be familiar with the resident, and subtle signs of clinical deterioration are easy to miss [9]. Recent evidence has shown that the quality of older adults’ gait, such as gait speed, variability, and balance, contains important information about their overall health status. For example, slower gait speed, shorter stride length, greater cycle-to-cycle gait variability, higher centre of pressure variability, and lower gait stability are associated with increased falls risk [10–14]. Moreover, gait speed changes measured over time in community-dwelling older adults are associated with adverse events and hospitalizations [15]. Advances in technology that allow longitudinal repeated monitoring of gait offer an opportunity to improve health status monitoring, but the need for an algorithm that detects clinically meaningful changes in gait (for example, that are predictive of a medical event) is a significant barrier to these monitoring technologies. A small number of longitudinal studies have been able to observe changes in gait preceding falls, diagnosis of a urinary tract infection or delirium in older adults [16–18]. However, at present, there is limited evidence that changes in gait can be detected reliably in advance of a deterioration in health. Similarly, few studies have tracked changes in gait after a hospital visit, to confirm that these changes stabilize in line with health stabilization.

The first aim of this study was to examine whether a change in gait in people with dementia receiving institutional care is observable before and after unplanned hospital visits. To address this aim, we completed a secondary data analysis using an observational dataset of gait measures captured repeatedly over time through pose-tracked videos. Within this dataset, we identified participants who had experienced an unplanned hospital visit and calculated the rate of change of gait measurements, prior to and after their hospital visit. The second aim was to determine if these observed changes in gait were associated specifically with hospitalization by comparing these gait changes to those in participants without a hospitalization event.

## Materials and methods

### Participants

This study is a secondary analysis of an existing dataset [12]. Participants (n = 54) were long-term care residents with a diagnosis of dementia who were temporarily residing in a dementia care unit to manage moderate to severe behavioural symptoms of dementia. The study protocol was approved by the Research Ethics Board of the University Health Network. Substitute decision makers were asked to provide written informed consent for all participants, while participants assented to all assessments. The inclusion criteria for the study were diagnosis of dementia, as established by a geriatric psychiatrist, and an ability to walk independently over a distance of 20 m. The only exclusion criterion was the use of a rolling walker with basket or seat which obscured the lower limbs in the video recordings.

The occurrence of a hospital visit was recorded retrospectively by reviewing participants’ medical records; the following information was extracted: the date and reason for the transfer to hospital recorded, and the date of their transfer back to the dementia care unit. Any participants with at least 3 days of recorded gait data pre-hospital visit were included in the pre-hospital visit analysis, and those with at least 3 days of recorded gait data post-hospital visit were included in the post-hospital visit analysis. Where there was more than one hospital visit, the first visit was selected and participant censored after the first visit. In the case where more than 100 days of gait data were available, a maximum of 100 days prior to and/or after the hospital visit was included to avoid including data very distant to the hospital event. The remaining participants had no hospital visit during the course of their study participation (n = 41).

As descriptive measures, the severity of dementia and behavioural symptoms were characterized using the Severe Impairment Battery score [19] and the Neuropsychiatric Inventory score [20].

### Gait recording

We used a vision-based system to record participants’ walking. The vision-based setup consisted of a Microsoft Kinect for Windows v2 (Microsoft, Redmond, Washington, USA) mounted on the ceiling of the unit’s hallway. A radio frequency identification system identified the participants, and custom-written software automatically turned on recording when a single figure was within view of the camera. The system captured walks as participants entered the hallway naturally, and thus there was variability in the number of walks captured per participant (dependent on their level of activity and whether they passed by the camera often). Gait data were collected from study entry until the participant was discharged from the unit.

### Gait variables

The three-dimensional joint motions during walks captured by the Kinect were extracted using the Kinect software development kit (SDK 2.0). Three categories of gait variables were calculated [12]: spatiotemporal (walking speed, step time and length), variability (coefficient of variation (CV) of step time and length), and mechanical stability (estimated margin of stability (eMOS) in medio-lateral direction). Step length was defined as the distance between the right and left ankles at foot strikes, step time as the time between the foot strike of one foot to the foot strike of the other foot, and gait speed as the displacement of the sacrum along the direction of movement divided by the elapsed time between the first and last step. The variability measures were calculated as the standard deviation of each gait parameter within each walking bout divided by the mean value. The average eMOS was calculated as the distance between estimated extrapolated centre of mass (sacrum position) to the ankle in medio-lateral direction during stance on each limb averaged over the recording. The ankle position in the medio-lateral direction was considered as the boundary of base of support. The x, y, and z axes were aligned with the medio-lateral, the vertical, and the anterior-posterior directions, respectively. Matlab software (Mathwork Inc, Natick, Massachusetts, USA) was used to calculate gait variables.

### Statistical analysis

We applied mixed effect models (linear growth models), which allow modeling of both fixed and random effects at two levels in repeated measures— level-1 (within-subject variability, usually over time) and level-2 (between-subject variability), that are particularly useful when the number of repeated measurements over time differs between participants [21]. In this analysis, separate models were created for each gait measure, with the gait measure as the dependent variable. We used the results of these models to answer our two research questions. First, to describe longitudinal gait changes before and after the hospital visit on an individual level, we examined each time series of gait measures and calculated the mean and standard deviation of the slope of gait measures separately in participants before, after, and without hospitalization. These slopes were visualized on spaghetti plots. Second, linear mixed models were used to investigate whether gait changes over time before a hospital visit differed from gait changes over time in participants without a hospital visit. Each of these models included a participant-level random intercept and fixed effects for time, hospital visit group, and hospital visit group x time interaction; age and sex were included as covariates in each model. R-software was used for all statistical analyses and the significance level was set at 0.05.

## Results

### Participants

The study participants are described in Table 1. All participants had moderate to severe dementia with Severe Impairment Battery score (mean ± standard deviation) of 28.5 ± 14.1 and moderate to severe behavioral symptoms of dementia (Neuropsychiatric Inventory score of 51.7 ± 21.2).

**Table 1:** Study participant demographics and mean ± standard deviation of gait variables

Demographic variables	Total (mean ± SD)	No hospital visit (mean ± SD)	Unplanned hospital visit (mean ± SD)
	n = 54	n = 41	n = 13*
Age (year)	76.4 ± 7.9	76.6 ± 7.4	75.8 ± 9.5
Sex (female n (%))	24 (44.4)	18 (43.9)	6 (46.1)
Height (cm)	163.6 ± 14.2	163.5 ± 15.2	164.0 ± 10.7
Weight (kg)	66.6 ± 12.8	66.2 ± 13.6	68.1 ± 10.3
Time in study (days)	48.2 ± 23.5	48.3 ± 23.7	48.1 ± 23.7
**Gait variables (averaged across full study period)**			
Number of walks captured	83.4 ± 70.4	88.2 ± 75.6	62.6 ± 46.9
Range (min, max)	8, 306	8, 306	13, 153
Gait speed (m/s)	0.52 ± 0.16	0.52 ± 0.16	0.49 ± 0.17
Step time (s)	0.62 ± 0.13	0.62 ± 0.13	0.63 ± 0.15
Step length (cm)	32.3 ± 9.9	32.6 ± 9.8	31.0 ± 10.5
Step time variability (%)	0.21 ± 0.22	0.21 ± 0.21	0.23 ± 0.23
Step length variability (%)	0.25 ± 0.23	0.25 ± 0.23	0.28 ± 0.25
eMOS (cm)	6.5 ± 2.8	6.3 ± 2.7	7.3 ± 3.2

*The hospitalization group provide information for all 13 participants who had unplanned hospital visits including 7 with gait data prehospital only, 4 with gait data before and after hospital, and 2 with gait data after the hospital only (total of 13).

Thirteen (24.1%) participants had at least one unplanned hospital visit over the stay (either an emergency department visit or acute care hospitalization). Three were due to fall-related injury and two due to other injury (not related to falls). Ten individuals visited the emergency department, three were hospitalised and one died (Supplemental Table 1). Of the thirteen participants with hospital visits, 11 had recorded walks before the hospital visit (7 with walks before only, and 4 with walks both before and after hospital visit), while 2 had walks only after the hospital visit.

### Descriptive analysis of changes in gait pre- and post-hospitalization

**Fig. 1:**
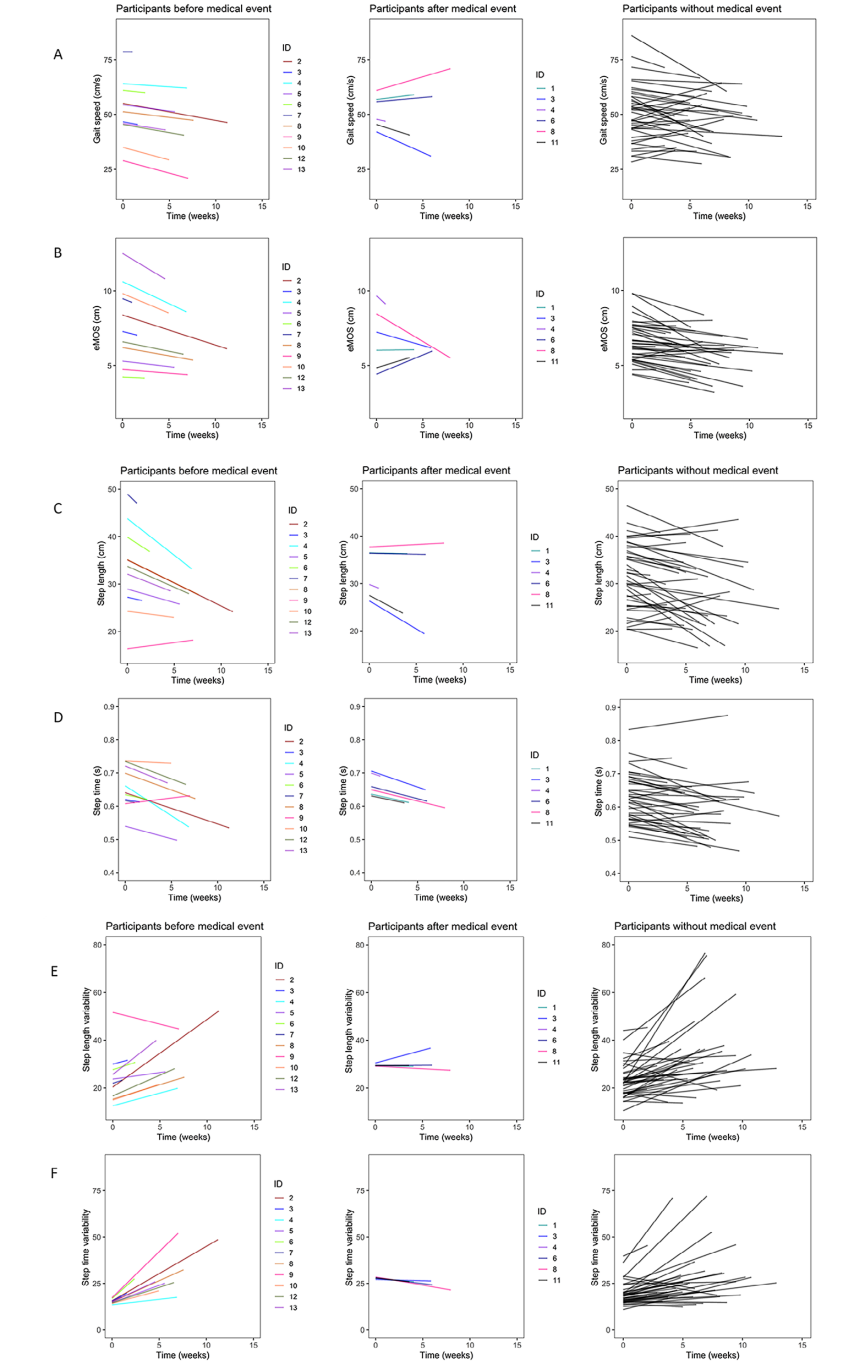
Change in spatiotemporal and stability measures of gait over time before hospital visit (column 1), after hospital visit (column 2) and in those who did not have a hospital visit (column 3). Gait measures depicted are **A**) gait speed, **B**) average eMOS **C**) step length, **D**) step time, **E**) coefficient of variability for step length and **F**) coefficient of variability for step time

Several gait measures showed a pattern of worsening gait prior to hospitalization (Table 2). For example, step length decreased for most participants in the time preceding hospitalization (-0.85 ± 0.22 cm/week (average slope ± standard deviation); Fig. 1C). The average eMOS had a decreasing slope (-0.17 ± 0.06 cm/week; Fig. 1B) and step time variability increased over time before hospitalization (2.51 ± 0.78%/week; Fig. 1F). Gait speed (Fig. 1A), step time (Fig. 1D), step length variability (Fig. 1E) had average slopes that were not statistically different from zero.

**Table 2:** Gait changes over time in participants before and after a hospital visit, and in those without a hospital visit

	Before hospital visit n = 11				After hospital visit n = 6				No hospital visit n = 41			
	Slope ± SD	t	p	df	Slope ± SD	t	p	df	Slope ± SD	t	p	df
**Gait speed (cm/s/week)**	-0.65 ± 0.32	-2.01	0.387	0.64	-0.33 ± 0.72	0.46	0.661	6.65	-0.36 ± 0.28	-1.28	0.208	34.60
**Step time (s/week)**	-0.007 ± 0.005	-1.60	0.161	6.03	-0.008 ± 0.007	-1.15	0.258	36.69	-0.005 ± 0.002	-2.79	**0.011**	22.57
**Step length (cm/week)**	-0.85 ± 0.22	-3.82	**0.003**	10.06	-0.50 ± 0.40	-1.24	0.247	8.35	-0.42 ± 0.15	-2.81	**0.008**	34.77
**Step time variability (%/week)**	2.51 ± 0.78	3.22	**0.018**	5.94	-0.53 ± 0.91	-0.58	0.588	4.65	1.26 ± 0.41	3.04	**0.005**	27.00
**Step length variability (%/week)**	1.32 ± 0.84	1.56	0.162	7.19	0.14 ± 0.92	0.16	0.879	11.31	1.43 ± 0.45	3.20	**0.003**	33.55
**eMOS (cm/week)**	-0.17 ± 0.06	-2.73	**0.014**	16.84	0.11 ± 0.16	-0.69	0.515	5.73	-0.12 ± 0.04	-3.09	**0.005**	24.87

Similar changes over time in gait variables were also seen in those without a hospitalization (Table 2), with decreased step length (-0.42 ± 0.15 cm/week) and average eMOS over time (-0.12 ± 0.04 cm/week), and increased step time variability (1.26 ± 0.41%/week). In addition, reductions in step time (-0.005 ± 0.002 s/week) and step length variability (1.43 ± 0.45%/week) over time were also recorded.

In the smaller cohort of those with data after a hospitalization event, the average slope for all gait variables was not statistically different from zero.

### Gait changes in those with and without a hospital visit

Pre-hospital visit gait changes in participants with a hospital visit were compared with gait changes in those without a hospital visit using linear mixed models that included age and sex as covariates. There were several differences between groups, with shorter step times and step length, increased step length and time variability, and reduced eMOS in the hospital visit group (Table 3). There were two significant group ✕ time interactions: gait speed (-0.85 ± 0.38 cm/s/week; p = 0.025) and step length (-0.54 ± 0.21 cm/week; p = 0.012) indicating a difference in trajectory (more rapid decline) in these gait measures preceding a hospital visit compared to those without a hospital visit, after adjusting for age and sex.

**Table 3:** Longitudinal gait changes before hospital visit in patients grouped by whether they had an unplanned hospital visit, adjusted for age and sex

Gait variables Before hospital visit		Estimate	SD	df	t-value	p-value
**Gait speed (cm/s/week)**	Group	-0.15	0.30	36	-0.49	0.63
	Time	-5.7	1.01	2700	-5.6	**< 0.001**
	Group X Time	-0.85	0.38	910	-2.2	**0.025**
**Step time (s/week)**	Group	-0.0064	0.0018	31	-3.5	**< 0.001**
	Time	0.013	0.0087	1300	1.5	0.12
	Group X Time	-0.00075	0.0031	270	-0.24	0.81
**Step length (cm/week)**	Group	-0.35	0.15	35	-2.38	**0.023**
	Time	-3.2	0.58	2000	-5.6	**< 0.001**
	Group X Time	-0.54	0.21	640	-2.5	**0.012**
**Step time variability (%/week)**	Group	1.2	0.31	35	3.8	**< 0.001**
	Time	-3.2	1.5	510	-2.2	**0.030**
	Group X Time	0.79	0.54	210	1.5	0.14
**Step length variability (%/week)**	Group	1.4	0.43	36	3.3	**0.002**
	Time	-1.6	1.6	1000	-0.97	0.33
	Group X Time	0.18	0.62	560	0.29	0.77
**eMOS (cm/week)**	Group	-0.11	0.035	21	-3.2	**0.005**
	Time	0.72	0.19	780	3.9	**< 0.001**
	Group X Time	-0.077	0.064	130	-1.19	0.24

## Discussion

This study aimed to determine whether people with severe dementia had a measurable change in gait prior to an unplanned hospital visit and whether this gait change differed from those who did not have an unplanned hospital visit. Study participants who were hospitalized had declines in step length and stability, and increasing step time variability in the period preceding the hospitalization. However, these changes were also observed over time in individuals who were not hospitalized. In age and sex-adjusted models, the slopes of gait speed and step length declined more over time prior to the hospital visit than in those without a hospital visit.

Our results are consistent with previous studies that found a relationship between gait measures, in particular gait speed, and future health outcomes. Studies with a single assessment of gait speed at baseline have shown that those with slow gait are at increased risk of emergency/hospital visits and death over a follow-up period of years [15, 22–24]. Fewer studies have examined longitudinal changes in gait or the relationship between gait changes and outcomes over shorter time periods. Studies with two or more gait speed assessments over time have also showed that slowing is a risk factor for adverse health outcomes, although it is less clear whether slowing is as important as slow gait at baseline [25]. In community-dwelling older adults, an average annual decline in gait speed of -0.015 m/s was observed in those who were hospitalized [26]. This is a much smaller magnitude of change compared to that observed over a short period of time in our frail, at-risk cohort. Changes in gait occurring over a short period of time before a medical event may be due to delirium, which has been associated with motor deficits and balance impairment [27]. Notably, a recent study found that gait slowing in LTC residents was associated with an increase in falls, but not urinary tract infections or delirium; however, this change was again assessed over a period of 18 months rather than immediately before the event [28].

While several gait variables were observed to have worsened over time, many of these observed changes were not specific to participants with a hospital visit, as changes were also seen in those who were not hospitalized. There are several possible explanations for this finding. It is possible that some participants experienced minor medical events (falls, injuries, infections, adverse medication events) over the course of the study that were treated without the need for a transfer to hospital. These medical events would not have been captured in our study and may explain why some participants without hospitalization also demonstrated gait decrements. Admission to a tertiary dementia care unit may also have contributed to some decline in gait across the whole sample independent of an unplanned hospitalization, due to the change in environment or adjustments to medications [13]. There was also variability between participants in the number of walks captured during the study. We used an observational design that required participants to walk past the research camera to have their gait measured. This has the advantage of being able to capture natural, everyday gait, and a large number of walks repeatedly over time, which is not possible with laboratory or clinic-based assessments [29]. A disadvantage of this approach is that participants who are unwell may reduce their motor activity and thus fail to have these changes in mobility captured by the recording system. The small number of participants (n = 13) with an event also impacted on the power of our statistical analysis to detect differences between those who were hospitalized and not hospitalized. Larger samples who are well characterized in terms of their medical status over time are needed for future definitive studies.

In the small number of participants who returned from hospital, there was no further gait decline. Previous studies have examined change in gait after hospitalization in community-dwelling older adults and shown that in general, hospitalization accelerates decline in gait speed, although the magnitude of these changes are small [26, 30], while short-term improvements in gait after hospitalization for heart failure are a good prognostic sign [31]. Given the small number of participants with data both pre- and post-hospital visit, we were not able to draw any specific conclusions about the impact of the hospital visit as an intervention. However, in general, it seems that with appropriate management for a medical event, accelerated loss of mobility can be arrested even in a frail cohort with advanced dementia. Another possibility is a “survivor effect,” with those who did return from hospital representing a more robust group.

While there was a significant difference in decline in gait speed and step length between those who were and were not hospitalized, we are not able to determine if the medical event could have been predicted based on the gait changes, or whether the hospitalization could have been prevented with earlier intervention or treatment provided on site. The small sample size is an important limitation, and there were only 4 participants with both before and after hospital visit data, which prevented us from drawing direct conclusions about the impact of the hospital visit on gait changes. In addition to the small number of participants, there was also variability in the number of recorded walks between participants related to factors such as their degree of mobility impairment and symptoms such as motor agitation and apathy. Our video-based method for repeated markerless motion capture and gait assessment does not track people using walkers, thus our results are not generalizable to people who regularly use gait aids. Our cohort was drawn from a population of older adults with dementia and behavioural and psychological symptoms of dementia and may not be representative of the long-term care population as a whole. Finally, by using hospital visits as the marker for a medical event, we have not captured medical events that were identified and treated on the unit without transfer to hospital. These pilot results will help to direct future studies with prospective monitoring of health status to better capture medical events of importance and short-term observable changes in gait.

## Conclusions

The gait of a cohort of older adults with dementia became more variable and more unsteady in the short time before an unplanned hospital visit. Gait speed and step length declined more quickly before a hospital visit compared to those who were not hospitalized. Future studies with larger, well-characterized study samples and prospective monitoring of health status are needed to identify clinically meaningful changes in gait associated with a deterioration in health.

### Electronic supplementary material

Below is the link to the electronic supplementary material.

Supplementary Material 1

## Data Availability

This study consists of secondary analysis of an existing video recording dataset (Medizadeh et al. 2019) [12]. The video dataset is not publicly available as the participants did not consent to publish their video recordings. Aggregate, de-identified data are, however, available from the authors upon reasonable request.

## Abbreviations

LTC: long–term care
CV: coefficient of variation
eMOS: estimated margin of stability
