# Supplementary material for: A pilot observational study of gait changes over time before and after an unplanned hospital visit in long-term care residents with dementia
Source: BMC Geriatr. 2023 Nov 8;23:723. doi: 10.1186/s12877-023-04385-0 (PMC10634101; doi:10.1186/s12877-023-04385-0)
Supplement: Supplementary file 1 — Supplementary Material 1 [file 12877_2023_4385_MOESM1_ESM.docx]

Supplemental Table 1: Details of first hospitalization events for 13 participants

| **ID** | **Total walks** | **Days in study** | **Day of hospital visit #** | **Days in hospital** | **Gait data before event (# days)** | **Gait data after event (# days)** | **Reason** |
| --- | --- | --- | --- | --- | --- | --- | --- |
| 1 | 153 | 29 | 1 | 1 | No (0) | Yes (29) | Finger injury |
| 2 | 149 | 80 | 80 | 9 | Yes (80) | No (0) | Hip fracture |
| 3 | 47 | 47 | 7 | 1 | Yes (7) | Yes (40) | Generalized weakness |
| 4 | 82 | 50 | 47 | 1 | Yes (47) | Yes (3) | Bright red blood in stool |
| 5 | 51 | 40 | 42 | 1 | Yes (40) | No (0) | Febrile and loss of consciousness |
| 6 | 58 | 51 | 10 | 3 | Yes (10) | Yes (38) | Pulmonary embolism |
| 7 | 24 | 8 | 23 | 1 | Yes (8) | No (0) | Urinary tract infection |
| 8 | 24 | 65 | 17 | 1 | Yes (17) | Yes (48) | Fall and laceration to lip |
| 9 | 42 | 50 | 57 | 1 | Yes (50) | No (0) | Small bowel obstruction |
| 10 | 106 | 110 | 32 | 1 | Yes (32) | No (78) | Pneumonia, shortness of breath, episode of decreased level of consciousness |
| 11 | 13 | 26 | -7* | 1 | No (0) | Yes (26) | Fall and head injury |
| 12 | 18 | 47 | 58 | 1 | Yes (47) | No (0) | Injury on face,  fall and head injury |
| 13 | 47 | 33 | 33 | 3 | Yes (33) | No (0) | Respiratory illness |

#Relative to starting date of study. *Hospital visit occurred 7 days prior to recruitment into study.
